# GETNet: Group Normalization Shuffle and Enhanced Channel Self-Attention Network Based on VT-UNet for Brain Tumor Segmentation

**DOI:** 10.3390/diagnostics14121257

**Published:** 2024-06-14

**Authors:** Bin Guo, Ning Cao, Ruihao Zhang, Peng Yang

**Affiliations:** 1 College of Information Science and Engineering, Hohai University, Nanjing 210098, China; drbinguo@163.com; 2 College of Computer and Information Engineering, Xinjiang Agricultural University, Urumqi 830052, China; dlstudyzrh@163.com (R.Z.); yangpeng990625@163.com (P.Y.)

**Keywords:** brain tumor segmentation, MRI, medical image, deep learning, Transformer

## Abstract

Currently, brain tumors are extremely harmful and prevalent. Deep learning technologies, including CNNs, UNet, and Transformer, have been applied in brain tumor segmentation for many years and have achieved some success. However, traditional CNNs and UNet capture insufficient global information, and Transformer cannot provide sufficient local information. Fusing the global information from Transformer with the local information of convolutions is an important step toward improving brain tumor segmentation. We propose the Group Normalization Shuffle and Enhanced Channel Self-Attention Network (GETNet), a network combining the pure Transformer structure with convolution operations based on VT-UNet, which considers both global and local information. The network includes the proposed group normalization shuffle block (GNS) and enhanced channel self-attention block (ECSA). The GNS is used after the VT Encoder Block and before the downsampling block to improve information extraction. An ECSA module is added to the bottleneck layer to utilize the characteristics of the detailed features in the bottom layer effectively. We also conducted experiments on the BraTS2021 dataset to demonstrate the performance of our network. The Dice coefficient (Dice) score results show that the values for the regions of the whole tumor (WT), tumor core (TC), and enhancing tumor (ET) were 91.77, 86.03, and 83.64, respectively. The results show that the proposed model achieves state-of-the-art performance compared with more than eleven benchmarks.

## 1. Introduction

A brain tumor may cause symptoms such as headache, dizziness, nausea and vomiting, lethargy, weakness, and optic disc edema [1]. If the tumor is large and compresses the optic nerve, decreased and blurred vision may occur [2]. Generally, tumors in the brain are more serious than those in other parts of the body. Benign and malignant tumors can continue to grow, increasing intracranial pressure [3]. Additionally, they can compress brain tissue and affect brain function, having a greater impact. Treatment plans for brain tumors are generally developed through medical imaging. Common medical imaging methods include X-ray imaging, computed tomography (CT), and magnetic resonance imaging (MRI) [4]. Compared with CT, MRI can obtain tomographic images in any direction, which helps display the anatomical relationship between tissue structures, clarify the origin and scope of lesions, and accurately diagnose the disease. It is safer to avoid the radiation damage that can be caused by traditional imaging methods, such as CT and X-ray [5]. MRI is widely used in clinical practice because it does not use radiation and produces high-resolution soft tissue and multisequence imaging. Although MRI is very helpful in the treatment of brain tumors, segmentation by hand is subject to human error, and evaluations vary among radiologists, leading to inconsistent results.

Deep learning can automatically learn useful features from many medical images [6], such as brain tumor shape, size, and boundary information, which are important in brain tumor segmentation and disease analysis. Convolutional neural network (CNN) models based on deep learning have advantages in image processing [7]. Convolution and pooling layers are used in CNNs. The convolution layer is responsible for extracting image features, and the pooling layer is used to greatly reduce the number of dimensions. Unlike CNNs, which are mainly used for feature extraction, classification, and the regression of input images, a fully convolutional network (FCN) adopts a full convolution structure and can adapt to variable-size input images. Thus, FCNs are widely used in image segmentation tasks. UNet is an excellent model that performs well in medical image segmentation. However, MRI images generally contain depth information, which is not fully utilized by the traditional 2D UNet; thus, 3D UNet was developed and has been popular for many years. Many researchers have developed excellent methods based on 3D UNet to complete segmentation tasks in the case of brain tumors. One of the weaknesses of 3D UNet is its extraction capability in terms of long-distance information, despite many attempts, such as atrous spatial pyramid pooling (ASPP), to expand the receptive field. Transformer deep learning methods, have achieved good results in natural language processing (NLP) and have been introduced into image processing to address this challenge. The Pyramid Vision Transformer (PVT), Swin Transformer, and Volumetric Transformer (VT-UNet) based on Transformer can capture the dependencies between different features through a self-attention mechanism, especially for long-distance dependencies, which has obvious advantages in image processing [8]. One of the challenges with Transformer is related to its focus on local details. We propose GETNet to improve the segmentation of brain tumor images to address the challenge of fusing global features and local details.

In this paper, we focus on integrating modules that extract local features with blocks that capture long-distance relationships. The main contributions of our work are as follows:

- We proposed a new GETNet for brain tumor segmentation which combined 3D convolution with VT-UNet to comprehensively capture delicate local information and global semantic information and improve brain tumor segmentation performance.
- We developed a GNS block between the VT Encoder Block and the downsampling module to enable the Transformer architecture to obtain local information effectively.
- We designed an ECSA block in the bottleneck layer to enhance the model for detailed feature extraction.

This paper is organized as follows: related work is described in Section 2. The materials and methods are presented in Section 3. In Section 4, comparison results and ablation experiments are presented and analyzed. Finally, Section 5 provides the discussion and conclusion.

## 2. Related Work

### 2.1. Deep-Learning-Based Methods for Medical Image Segmentation

In recent years, research on image analysis and segmentation has made breakthroughs with the proposal of deep learning methods, as represented by CNNs [9]. CNNs can learn representative image features by continuously iterating model parameters and then constructing a model for subsequent segmentation tasks. The block-based CNN method uses images as the network input and adopts image classification to replace pixel classification in the image. Because traditional CNNs use a sliding window frame based on images for image segmentation, the overlap between adjacent image blocks will lead to repeated convolution calculations during training, increasing calculation time and reducing efficiency. Long et al. [10] proposed using an FCN to classify images at the pixel level to solve the problem of image segmentation at the semantic level and achieved good results. An FCN can classify images at the pixel level and has no limit in terms of input image size; it can reduce the number of computations and improve the efficiency of segmentation compared with traditional CNNs. However, this approach lacks spatial consistency and does not fully use contextual information. In 2015, Ronneberger et al. [11] proposed UNet, a U-shaped CNN for medical image segmentation, to address this challenge. Özgün Çiçek et al. [12] proposed 3D UNet, which extends the previous UNet by replacing all 2D operations with 3D operations. Applying three-dimensional depth information is helpful for improving the performance of brain tumor segmentation. Recently, many network variants have been proposed due to the success of 3D UNet. Many of these networks attempt to expand the receptive field to extract global features. DeepLabv1 was proposed by Chen et al. [13] to ensure that feature resolution is not reduced and that the network has a larger receptive field. DeepLabv2, DeepLabv3, and DeepLabv3+ were subsequently developed by Chen et al. [14,15,16]. Chen et al. [17] designed DMFNet to construct multiscale feature representations via 3D dilated convolutions. Xu et al. [18] proposed a network to capture multiscale information using 3D atrous spatial pyramid pooling (ASPP). Jiang et al. [19] developed AIU-Net with the ASPP module to expand the receptive field and increase the width and depth of the network. Parvez Ahmad et al. [20] designed RD^2^A 3D UNet to preserve more contextual information of small sizes. A multiscale feature extraction module was developed by Wang et al. [21] to extract more receptive fields and improve the ability to capture features with different scales. The E1D3 network was introduced by Syed Talha Bukhari et al. [22] to perform effective multiclass segmentation. There are one-encoder and three-decoder fully convolutional neural network architectures where each decoder segments one of the hierarchical regions of interest (WT, TC, and ET) in the E1D3 network. Parvez Ahmad et al. [23] suggested that multiscale features are very important in MS UNet. Wu et al. [24] proposed SDS-Net to enhance segmentation performance. Local space with detailed feature information was designed by Chen et al. [25] to increase the detailed feature awareness of voxels between adjacent dimensions. MonaKharaji et al. [26] incorporated residual blocks and attention gates to capture emphasized informative regions. Regarding the BraTS dataset, the final segmentation results are divided into three parts: whole tumor (WT), tumor core (TC), and enhancing tumor (ET). They have an inclusive relationship, meaning that WT encompasses both TC and ET, with ET being included within TC. Using multiscale receptive fields to extract features for the three regions has advantages over using only a single receptive field. Despite acknowledging that considerable research has been carried out concerning the capture of contextual information or the expansion of the receptive field, the effective fusion of local feature information and the long-distance relationships between features is also crucial for multi-subregion segmentation of brain tumors (WT, TC, and ET).

### 2.2. Attention-Based Module for Medical Image Segmentation

An attention mechanism is a weighted change in target data. It is widely used in clustering learning, reinforcement learning, image processing, and speech recognition. An attention mechanism based on deep learning that imitates the human visual system automatically adopts some visual areas that need to be focused on and can improve the effectiveness of related learning tasks. Both spatial attention [27] and channel attention mechanisms can be used to recalibrate the characteristic information of the input data. They generally utilize a global pooling operation to obtain richer global information. One of the differences is that the channel attention mechanism performs global pooling layer by layer along the channel direction. In contrast, the spatial attention mechanism focuses on the feature information at a different location. Recently, many researchers have focused on multiscale and contextual information. Zhou et al. [28] designed attention mechanisms for learning contextual and attentive information. Zhang et al. [29] constructed the SMTFNet to aggregate global feature information. Zhao et al. [30] developed MSEF-Net to adapt a multiscale fusion module. Liu et al. [31] proposed MSMV-Net while considering the strengths of multiscale feature extraction. Wang et al. [32] proposed a multiscale contextual block to focus on spatial information at different scales. Self-attention [33] can establish a global dependency and expand the receptive field of an image, which is the foundation of Transformer methods. The above are all based on convolutional approaches. Local convolutional operations are limited by the size of the convolutional kernel, which results in a weaker perception of global features. Transformers, through self-attention, can capture dependencies at various positions; the receptive field for global features is relatively large, increasing the richness of global information and allowing for the capture of more information from medium to large targets.

### 2.3. The Transformer-Based Module for Medical Image Segmentation

The excellent performance of the Transformer in natural language processing tasks fully demonstrates its effectiveness. The breakthrough of Transformer networks in NLP has stimulated interest in applying them to computer vision tasks. Alexey Dosovitskiy et al. [34] proposed the Vision Transformer (ViT) to capture long-range dependencies in images through a global attention mechanism, a milestone in the application of Transformers to computer vision. Wang et al. [35] developed the Pyramid Vision Transformer (PVT) to generate multiscale feature maps for intensive prediction tasks. Liu et al. [36] presented the Swin Transformer, which captures global feature information via self-attention. Many researchers have investigated pure Transformers, such as the Volumetric Transformer Net (VT-UNet) [37]. Their advantage is that the encoder benefits from the self-attention mechanism by encoding local and global features simultaneously, while the decoder uses parallel self-attention and cross-attention to capture fine details for boundary refinement. Ali Hatamizadeh et al. [38] utilized a Transformer as an encoder to learn sequence representations of the input volume and effectively capture global multiscale information. However, one of the disadvantages of pure Transformers is that they only focus on global contextual information and address local details less. As stated earlier, in the BraTS dataset, the final segmentation results are divided into three parts: whole tumor (WT), tumor core (TC), and enhancing tumor (ET). In the task of brain tumor segmentation, the three indicators, as well as the boundary information, global information, and local information, need to be used together to enhance the final segmentation results. Recently, considerable research on brain tumor segmentation based on the fusion of Transformers and CNNs has been conducted. Jia et al. [39] proposed using BiTr-UNet, a combined CNN–Transformer network, to achieve good performance on the BraTS2021 validation dataset. TransBTS was developed by Wang et al. [40] to capture local 3D contextual information. Cai et al. [41] reported that Swin UNet can adequately learn both global and local dependency information in all layers of an image. Fu et al. [42] proposed HmsU-Net, a hybrid multiscale UNet based on the combination of a CNN and Transformer for medical image segmentation. Ao et al. [43] developed an effective combined Transformer–CNN network using multiscale feature learning. Ilyasse Aboussaleh et al. [44] designed 3DUV-NetR+ to capture more contextual information. Recently, hybrid architectures of CNNs and Transformers have been research hotspots. The further development of this research is very beneficial for improving performance in brain tumor segmentation.

## 3. Materials and Methods

### 3.1. Datasets and Preprocessing

The brain tumor segmentation challenge (BraTS) dataset [45,46] is a public medical image dataset used to research and develop brain tumor segmentation algorithms. The BraTS dataset integrates four MRI modalities: T1-weighted (T1), T2-weighted (T2), T1-enhanced contrast (T1ce), and fluid-attenuated inversion recovery (FLAIR). The BraTS2021 [47] dataset, consisting of data from 1251 patients for training and 219 patients for validation, is popular among researchers. Generally, 1251 cases all contain ground truths labeled by board-certified neuroradiologists, while the 219 ground-truth cases are hidden from the public; the results can be obtained only via online validation. Our training strategy included 80% and 20% of the BraTS2021 training data for training and validation, respectively. In addition, we uploaded our prediction results to the official BraTS platform (https://www.synapse.org/#) (accessed on 12 June 2024) for model evaluation.

**Figure 1:**
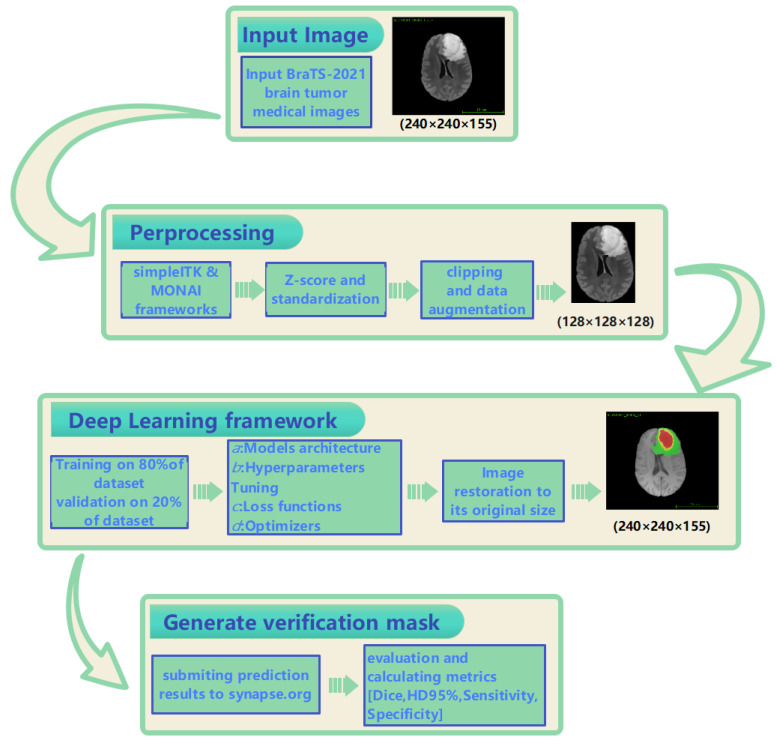
Procedural flowchart of the proposed GETNet.

In order to enable our network to segment brain tumor images normally, we first read the BraTS2021 dataset into our program in the preprocessing stage. After processing with simpleITK and MONAI, we used the Z-score method to standardize each image. Sequentially, we reduced the background as much as possible while ensuring that all of the brain was included and randomly re-cropped the fixed patch size of the image to 128 × 128 × 128. All of the intensity values were clipped to the 1st and 99th percentiles of the non-zero voxel distribution of the volume. In this research, we used rotation between −30 and 30, additive Gaussian noise of a centered normal distribution with a standard deviation of 0.1, blurring between 0.5 and 1, and the addition of a gamma transformation value between 0.7 and 1.5 as data augmentation techniques. The procedural flowchart of the proposed GETNet is depicted in Figure 1.

### 3.2. Implementation Details

Our network was constructed using Python 3.8.10 and PyTorch 1.11.0. A single NVIDIA RTX A5000 with 24 G memory and AMD EPYC 7551P were used during training. Table 1 shows that the initial learning rate was 1.00 × 10^−4^, with a batch size of 1. Cuda version cu113 was used. During training, Adam [48] was used to optimize our network. Unlike the case of hybrid loss, only ordinary soft Dice loss [49] was trained in our network. The input and output sizes were both 128 × 128 × 128.

### 3.3. Evaluation Metrics

Quantitative and qualitative analyses were carried out using evaluation metrics, including the Dice similarity coefficient (Dice) score [50], the Hausdorff distance (HD) [51,52], sensitivity, and specificity.

Dice is a measure of the similarity between two effects. It is used to measure the similarity between the results predicted through network segmentation and manual masks in image segmentation, and it which can be represented as follows:

(1) $$\mathrm{Dice}=\frac{2\mathrm{TP}}{2\mathrm{TP}+\mathrm{FP}+\mathrm{FN}}$$

where TP, FP, and FN represent true positive cases, false positive cases, and false negative cases, respectively.

HD represents the maximum distance between the predicted and real region boundaries. The smaller the value is, the smaller the predicted boundary segmentation error and the better the quality. HD95 is similar to the maximum HD, but it is calculated based on the 95th percentile of distances between boundary points in t and p. The purpose of using this measure is to mitigate the impact of a very small subset of outliers. HD can be represented as follows:

(2) $$\mathrm{HD}(P,T)=\max\{\sup_{t\in T\;}\mathrm{in}\;f_{p\in P}d\left(t,p\right),\;\sup_{p\in P\;}\mathrm{in}\;f_{t\in T}d\left(t,p\right)\}$$

where t and p represent the real region boundary and predicted segmentation region boundary, respectively. d(•) represents the distance between t and p. Sup denotes the supremum.

Sensitivity refers to as the true positive rate. It quantifies the accurate probability of complete positive detection. The sensitivity can be represented as follows:

(3) $$Sensitivity=\frac{TP}{TP+FN}$$

where *TP* and *FN* represent true positive cases and false negative cases, respectively. A higher sensitivity corresponds to a smaller discrepancy between glioma segmentation and the ground truth.

The *Specificity* represents the true negative rate, which reflects the probability of complete negative detection. The specificity can be represented as follows:

(4) $$Specificity=\frac{TN}{TN+FP}$$

where *TN* and *FP* represent true negative cases and false positive cases, respectively. The higher the specificity is, the smaller the difference between the segmentation and ground truth for normal tissue.

### 3.4. Methodology

#### 3.4.1. Network Architecture

**Figure 2:**
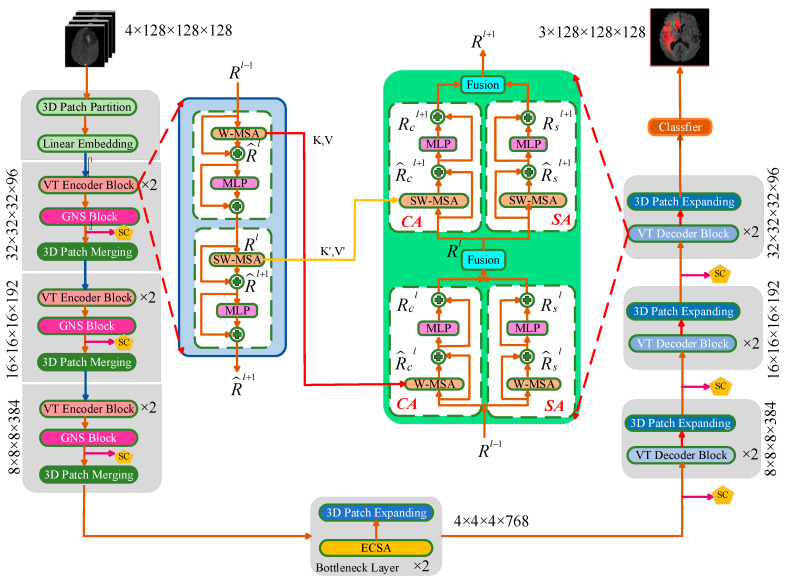
An illustration of the proposed GETNet for brain tumor image segmentation.

The effective integration of local features and global relationships is very helpful for improving the performance of brain tumor segmentation tasks. As shown in Figure 2, our network is a U-shaped architecture based on a Transformer with convolution operations. The encoder branch is on the left, the bottleneck layer is located at the bottom, and the decoder is on the right of the architecture. The encoder incorporates a 3D Patch Partition Block, Linear Embedding Block, VT Encoder Block, GNS Block, and 3D Patch-Merging Block. The 3D Patch Partition Block cuts the brain tumor images into nonoverlapping patches, and the Linear Embedding block maps the tokens to a vector dimension equal to the number of channels. The 3D Patch-Merging Block reduces the size of the image by half and doubles the number of channels, similarly to the process of pooling or convolution with a stride of 2 in a CNN. This operation is akin to downsampling and increasing the feature depth, contributing to the overall efficiency and effectiveness of the network architecture. After the 3D Patch-Merging operation, VT-UNet only changes the height and width, while the depth remains unchanged. To reduce the image dimensions and floating-point operations per second (FLOPs) and to prevent overfitting, changes were made to the height, width, and depth of our model.

The GNS Block addresses the issue of insufficient local features in feature extraction. A hierarchical representation is constructed by the VT Encoder Block from small patches, which are gradually merged with neighboring patches as the Transformer layers deepen to capture better features. The two modules in one decoder layer are the 3D Patch Expanding Block and the VT Decoder Block. Here, 3D Patch Expansion reshapes the image size along the spatial axis, doubling the image size and reducing the number of channels by half. The VT Decoder Block integrates high-resolution information from the encoder and low-resolution information from the decoder to recover features lost during downsampling and improve segmentation accuracy.

Notably, the VT Encoder Block, VT Decoder Block, and ECSA Block are used twice. The VT Decoder Block combines a self-attention block and cross-attention to improve the prediction quality. The SC Block is similar to the UNet skip connection, which establishes a bridge for information transmission between the encoding layer and the corresponding decoding layer. Specifically, the values of both K and V generated by the multi-head self-attention (W-MSA) of the VT Encoder Block are passed to the W-MSA of the VT Decoder Block. Similarly, the shifted window-based multi-head self-attention (SW-MSA) of the VT Encoder Block delivers K′ and V′ to the SW-MSA of the VT Decoder Block in the same way. The bottleneck layer has two modules: the 3D Patch-Expanding Block, whose function is the same as that of the 3D Patch Expanding of the decoder, and the ESCA block, which can capture detailed features with long-distance relationships of the bottom layer.

**Figure 3:**
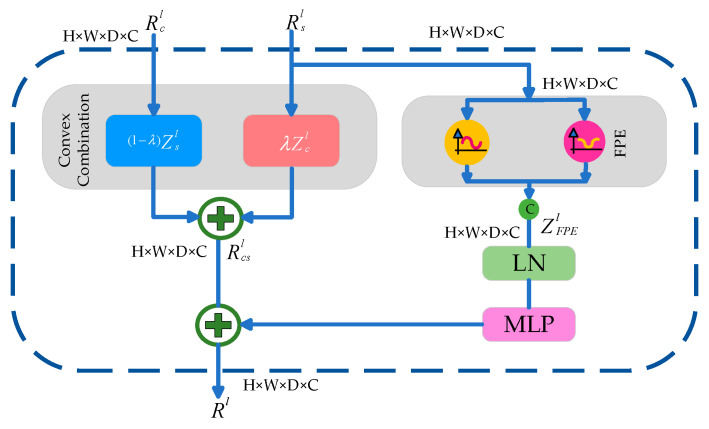
An illustration of the fusion sub-block.

The VT Encoder Block and VT Decoder Block employ attention layers with windows to important feature information when capturing long-distance dependencies between tokens. The attentions of W-MSA and SW-MSA in the VT Encoder Block and VT Decoder Block utilize tokens within the window to help with representation learning. In W-MSA, we uniformly divide the volume into smaller nonoverlapping windows. The tokens in adjacent windows of W-MSA cannot be seen by each other. In contrast, they can see each other by using the shifting window in SW-MSA, which facilitates the interaction of information between different windows, thereby guiding effective feature extraction. The VT Decoder Block can be divided into two parts: the left part is cross-attention (CA), and the right part is self-attention (SA). The fusion subblock, shown in Figure 3, merges the results of CA and SA and delivers them to the later layer. The fusion subblock comprises a convex combination, Fourier feature positional encoding (FPE), layer normalization (LN) [53], and a multi-layer perceptron (MLP) [54]. The dimensions of the input image are 4 × 128 × 128 × 128, and the classifier layer includes a 3D convolutional layer to map deep dimensional features to 3 × 128 × 128 × 128.

#### 3.4.2. Enhanced Channel Self-Attention Block (ECSA)

**Figure 4:**
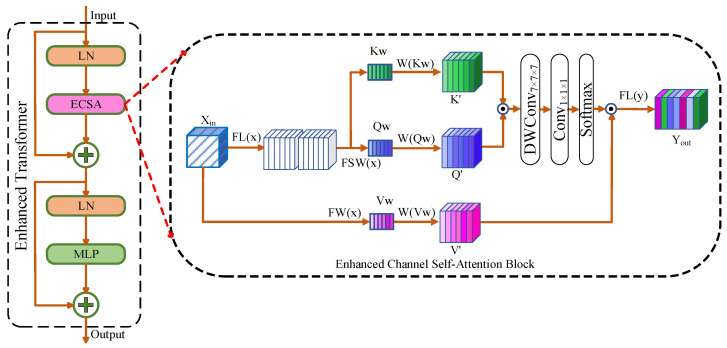
An illustration of the building blocks of the ECSA Block.

A diagram of the Enhanced Transformer and ECSA Block is shown in Figure 4. The bottom layer is the lowest in the network and has the smallest image size. However, it contains the richest semantic information. It is helpful to extract detailed features effectively from the bottleneck layer, which is important in terms of the brain tumor segmentation results. The Enhanced Transformer combines the advantages of global and local features, which is beneficial for extracting the details of image features and can be represented as follows:

(5) $$Z=\left(ECSA\left(LN\left(x\right)\right)+x\right)+MLP(LN\left(ECSA\left(LN\left(x\right)\right)+x\right))$$

where *x* denotes the input features. *LN* represents layer normalization. *MLP* is a multi-layer perceptron. *Z* is the result of the equation. The *ECSA* Block is an enhanced channel self-attention block.

The ECSA Block first extracts the channel weights, *Kw*, *Qw*, and *Vw*, of the image features. A weighted self-attention mechanism was developed to capture more effective global features. Depth-wise separable convolution [55] with large convolution kernels of 7 × 7 × 7 is used to ensure larger receptive fields, and it is then performed on each channel to obtain local features while minimizing information loss. Finally, all channels are aggregated using a 1 × 1 × 1 convolution before being output. The ECSA can be divided into three steps: calculating the weights, capturing the weighted global features, and fusing the local features.

First step: The calculation formula for the three weights can be described as follows:

(6) $$Qw=FSW(FL\left(x\right))$$

(7) $$Kw=FSW(FL\left(x\right))$$

(8) Vw=FW(x)

where *FL* denotes a linear operation. *FSW* can be calculated as follows:

(9) $$FW\left(x\right)=sigmoid(\left(FL\left(Relu\left(FL\left(AP\left(x\right)\right)\right)\right)\right))$$

(10) $$FSW\left(x\right)=sigmoid(\left(FL\left(Relu\left(FL\left(AP\left(x\right)\right)\right)\right)\right))$$

where *AP* denotes average pooling. *FL* represents a linear operation.

In the second step, the capture of the weighted global features is calculated as follows:

(11) $$Q^{\prime }=W((Qw),FL(x))$$

(12) $$K^{\prime }=W((Kw),FL(x))$$

(13) $$V^{\prime }=W((Vw),x)$$

where *W(•)*, which is a multiplication operation using the input data, can be represented as follows:

(14) $$Q^{\prime }=Qw\times FL(x)$$

(15) $$K^{\prime }=Kw\times FL(x)$$

(16) $$V^{\prime }=Vw\times x$$

In the third step, the fusion of the local features is calculated as follows:

(17) $$Y_{out}={FL((softmax(Conv_{1\times 1\times 1}(DWC_{7\times 7\times 7}(K}^{\prime }⊙Q\prime ))))⊙V\prime )$$

where *Y_out_* denotes the final result. *Conv*_1×1×1_ represents convolution with a 1 × 1 × 1 kernel. DWC_7×7×7_ is expressed as a depth-wise separable convolution with a 7 × 7 × 7 kernel. *FL* denotes a linear operation. ⊙ denotes the Hadamard product [56], which converts second-order mappings into third-order mappings.

#### 3.4.3. Group Normalization Shuffle (GNS) Block

**Figure 5:**
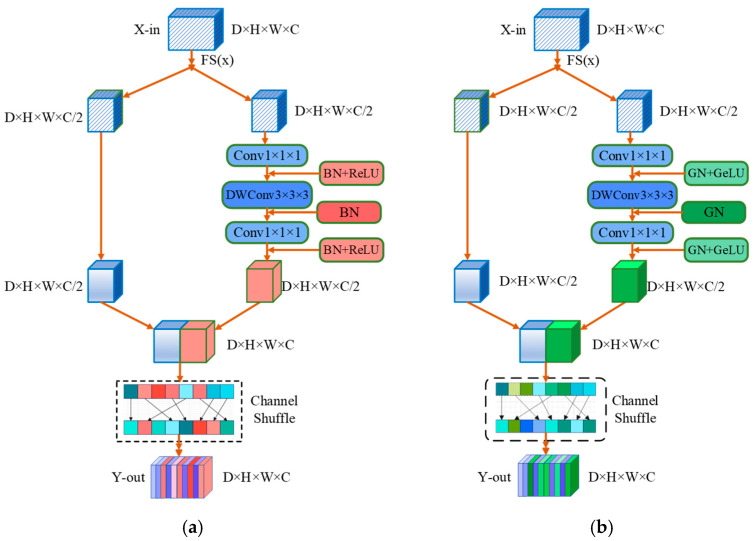
An illustration of the building blocks from a channel-wise perspective. (**a**) The shuffle block in ShuffleNetV2; (**b**) the GNS Block presented in this paper.

A diagram of the GNS Block is shown in Figure 5. Ma et al. [57] proposed ShuffleNetv2 to divide the input feature map into multiple subblocks and perform a shuffling operation on these subblocks. Shuffling operations typically involve rearranging the features between different subblocks to introduce more variation and diversity. This process helps the model better capture details and structures in images and improve the generalization ability.

Batch normalization (BN) has become an important component of many advanced deep learning models, especially in computer vision. BN normalizes layer inputs by calculating the average and variance in batch processing. The batch size must be sufficiently large, for BN to perform well. However, only small batches are available in some cases. Group normalization (GN) [58] is suitable for tasks that require a large amount of memory, such as image segmentation. GN calculates the mean and variance in each group channel-wise and is not related to or constrained by batch size. As the batch size decreases, GN performance is basically unaffected.

The rectified linear unit (ReLU) and Gaussian error linear unit (GeLU) [59] are the most common activation functions. ReLU is a very simple function that returns 0 only when the input is negative and returns the value of the input when the input is positive. Thus, it contains only one piecewise linear transformation. However, the ReLU output remains constant at 0 when the input is negative. This problem may lead to neuronal death, reducing the expression of the model. The GeLU function is a continuous S-shaped curve with a smoother shape than that of ReLU, and it can alleviate neuronal death to a certain extent. Inspired by the above, we utilized GN instead of BN and replaced ReLU with GeLU in our GNS Block to enable the communication of information between different channel groups and improve accuracy, which can be represented as follows:

(18) $$d=\mathrm{shuffle}(\mathrm{concat}\left(\mathrm{FS}\left(x\right),\;\mathrm{Con}v_{1\times 1\times 1}\left(\mathrm{DW}C_{3\times 3\times 3}\left(\mathrm{Con}v_{1\times 1\times 1}\left(\mathrm{FS}\left(x\right)\right)\right)\right)\right))$$

where FS(•) denotes the split operation, which divides the features of one channel into two channels on average. Conv_1×1×1_ represents a convolution with a 1 × 1 × 1 kernel. DWC_3×3×3_ is expressed as a separable convolution with a 3 × 3 × 3 kernel. The concat operation represents the concatenation of two sets of features. The shuffle operation is a channel shuffle operation.

## 4. Results and Discussion

### 4.1. Comparison with Other Methods

We compared eleven advanced models to evaluate the advantages of the proposed model. Two networks were compared for 2024, two for 2023, and two for 2022, in addition to five classic networks. The five classic networks were 3D UNet, Att-UNet, UNETR, TransBTS, and VT-UNet. There are six architecture variants based on basic UNet, and five are structures based on Transformer. In order to accurately validate the effectiveness of the model that we proposed, we sequentially compared it with different methods offline and online on BraTS2021. The offline results were obtained by running experiments on our servers, while the online results were obtained after uploading the model to the official BraTS platform and receiving the official results. We utilized five-fold cross-validation in our offline experiments. In Table 2, the offline results are presented for comparison with those of other methods. In the table, it can be observed that except for a slightly lower F1-score value, all other values are the highest. As shown in Table 3, we separately conducted a statistical significance analysis to compare different methods using the BraTS2021 dataset; the results were determined with a one-sided Wilcoxon signed rank test. Bold numbers indicate statistical significance (*p* < 0.05).

Inspired by the studies of Michael Rebsamen [61] and Snehal Prabhudesai [62], GETNet method was validated separately on the HGG dataset (293 cases), LGG dataset (76 cases), and a combination of the two. In Table 4, it can be seen that the Dice values of the HGG cases are relatively higher, followed by the mixed HGG and LGG values, and the LGG cases are slightly lower. In the BRATS dataset, due to the lack of representation of LGG samples, there was an inevitable performance decrease for the LGG data. There are typically no necrotic areas; hence, they exhibit significant differences in the appearance of the tumor core region compared to the HGG. Additionally, the appearance and size of the enhancing tumor region are also distinct. This impacts the network’s performance, and these differences can lead to suboptimal model performance in HGG segmentation.

**Figure 6:**
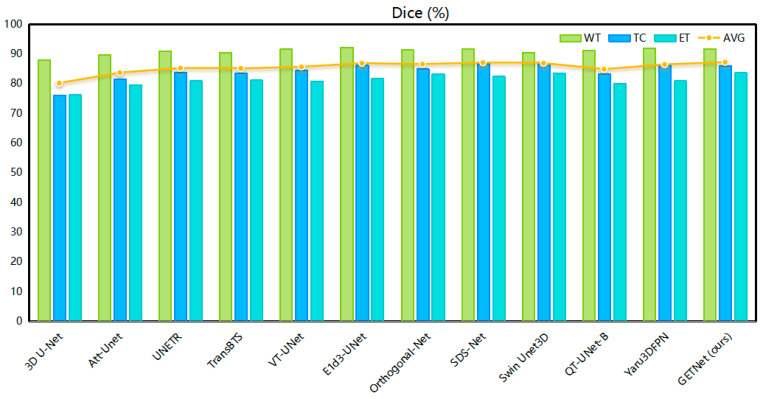
Comparison of the Dice results of different segmentation methods.

**Figure 7:**
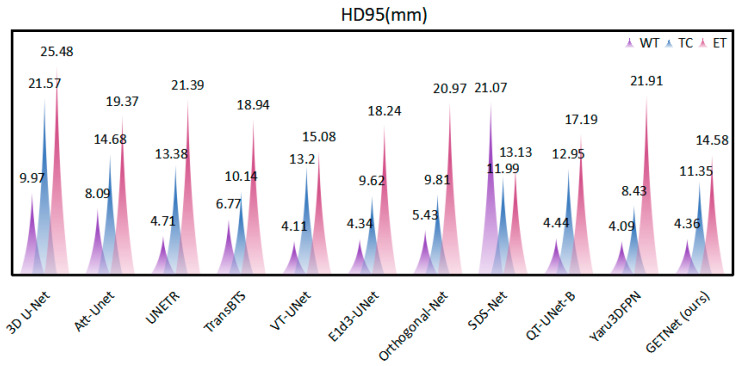
Comparison of the HD results of different segmentation methods.

Table 5 and Figure 6 and Figure 7 show that the Dice coefficient values of GETNet in the online validation for the whole tumor (WT), tumor core (TC), and enhancing tumor (ET) are 91.77, 86.03, and 83.64, respectively. The values of HD, shown in Table 5, are 4.36, 11.35, and 14.58 for the three tumor subregions (WT, TC, and ET), respectively. We used VT-UNet as the baseline, and our WT, TC, ET, and average Dice results increased by 0.11, 1.62, 2.89, and 1.55, respectively, when the values of HD95 were close to each other. From the results, it can be seen that the incorporation of convolution into the pure Transformer (which also has local characteristics) improved its operation.

**Figure 8:**
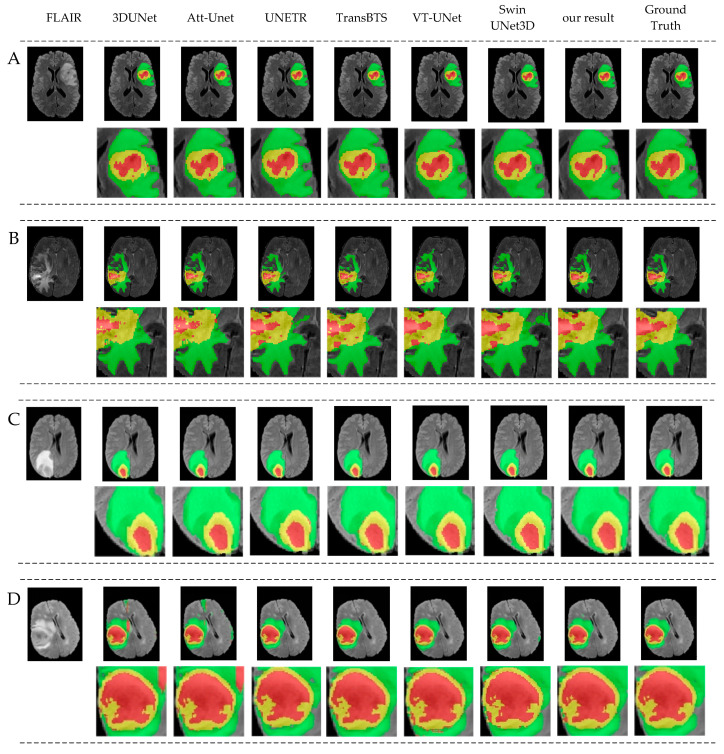

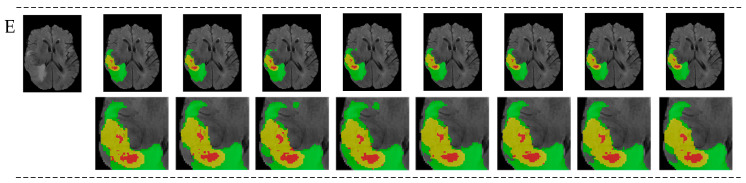
Visualization results for medical cases. From left to right: FLAIR, 3DUNet, Att-Unet, UNETR, TransBTS, VT-UNet, SwinUNet3D, the results segmented by GETNet, and the ground truth. (**A**–**E**) are five cases were randomly chosen on the BraTS2021 dataset. Green, yellow, and red represent WT, TC, and ET, respectively.

The results show that our network slightly improved in terms of TC and ET; that is, our network performs better than the baseline in small target segmentation. Compared to other networks, ours may not be the best on a single indicator, but our average results and ET values are the highest. Regarding the BraTS dataset, the final segmentation results are divided into three parts: whole tumor (WT), tumor core (TC), and enhancing tumor (ET). They have an inclusive relationship, meaning that WT encompasses both TC and ET, with ET being included within TC. If there is an emphasis on enhancing the focus on local detail features, the ET results are likely to improve. This indicates that our segmentation performance for small targets is the best among the compared networks, mainly due to the incorporation of local detail features. Figure 8 shows the visualization results of the GETNet model on the BraTS2021 dataset, in which five cases were randomly chosen. The medical cases, as shown in sequences A, B, C, D, and E of Figure 8, were segmented by GETNet. The figures from left to right are, respectively, FLAIR, 3DUNet, Att-Unet, UNetr, TransBTS, VT-UNet, SwinUNet3D, the results segmented by GETNet, and the ground truth. Green, yellow, and red represent WT, TC, and ET, respectively. In general, the results of GETNet are close to the labeled ground truth. Compared to the network with only convolutions, the results of our model are the best. Our network also performs better than the networks based on a Transformer. Overall, our architecture and modules achieved better results in relation to BraTS2021, providing a good basis for subsequent research.

### 4.2. Ablation Experiments

#### 4.2.1. Ablation Study of Each Module in GETNet

**Figure 9:**
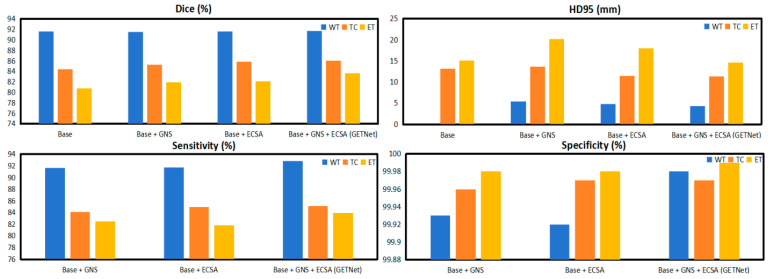
The results of the ablation study of each module in GETNet.

We conducted ablation experiments to verify the effects of different modules in this architecture. Table 6 and Figure 9 show the results of utilizing GNS and ECSA in GETNet, which improved the average Dice coefficient by 0.64 and 0.91, respectively. Empirically, we added both the GNS and ECSA, and all indicators improved. The results are 91.77, 86.03, 83.64, and 87.15 for WT, TC, ET, and the average Dice coefficient, respectively. The Hausdorff 95% (HD) values are 4.36, 11.35, 14.58, and 10.10 for WT, TC, ET, and the average HD, respectively.

The original plan was to use a pure Transformer, which does not include the local connectivity of convolution (capturing local features), shared weights (reducing the number of parameters), and sparse interactions (reducing the number of parameters and computational overhead). The module that we designed uses convolution within the pure Transformer architecture; thus, it captures local features. Table 6 shows that whether we add the GNS module alone, the ECSA module alone, or both, there is an improvement. The best results show that our network and all of the modules can be effectively applied to brain tumor segmentation tasks.

#### 4.2.2. Ablation Study of GN and GeLU in the GNS Module

**Figure 10:**
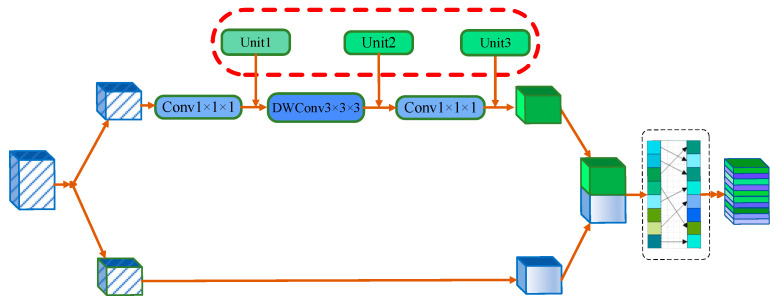
An illustration of building blocks in the channel-wise perspective of GNS.

To verify the effectiveness of replacing BN and ReLU with GN and GeLU, we conducted five sets of experiments. The results and experimental plan are shown in Table 7. Experiment A uses the original shuffle block of ShuffleNet V2. Unit 1, Unit 2, and Unit 3 can be seen in Figure 10, which represents BN, GN, BN + ReLU, and GN + GeLU placed at different positions.

In the experiment, the first combination in Unit 1 was GN, that in Unit 2 was GN + GeLU, and that in Unit 3 was GN, and the effect improved (Experiment E had the best result). Next, we attempted the case where Unit 1, Unit 2, and Unit 3 were all GN + GeLU, and the results improved compared to those of Experiment A but were worse than those obtained in Experiment E. If all were replaced with GN, the results could improve (Experiment C) compared to those of Experiment A, but they were similar to those of Experiment B. Naturally, we also replaced Unit 1, Unit 2, and Unit 3 (Experiment D) in Experiment E with GN, GN + GeLU, and GN, and the results worsened. The experimental plans were as follows:

A: Unit 1 → BN+ReLU; Unit 2 → BN; Unit 3 → BN+ReLU;

B: Unit 1 → GN+GeLU; Unit 2 → GN+GeLU; Unit 3 → GN+GeLU;

C: Unit 1 → GN; Unit 2 → GN; Unit 3 → GN;

D: Unit 1 → GN; Unit 2 → GN+GeLU; Unit 3 → GN;

E: Unit 1 → GN+GeLU; Unit 2 → GN; Unit 3 → GN+GeLU.

**Figure 11:**
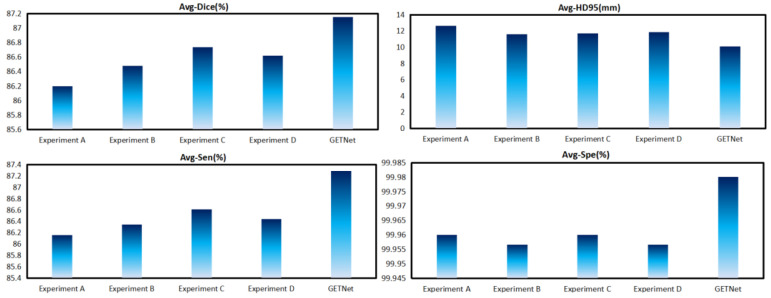
The results of the ablation study of GN and GeLU in the ECSA module.

The results are shown in Table 7 and Figure 11. We imitated the shuffle block and replaced the corresponding BN and ReLU with GN and GeLU, respectively, achieving good results. The results are 91.77, 86.03, 83.64, and 87.15 for WT, TC, ET, and the average Dice coefficient, respectively. The results of Experiment E are still the best. These results exceed those of the shuffle block, indicating that our improvement is effective.

#### 4.2.3. Ablation Study of the Convex Combination in the ECSA Module

**Figure 12:**
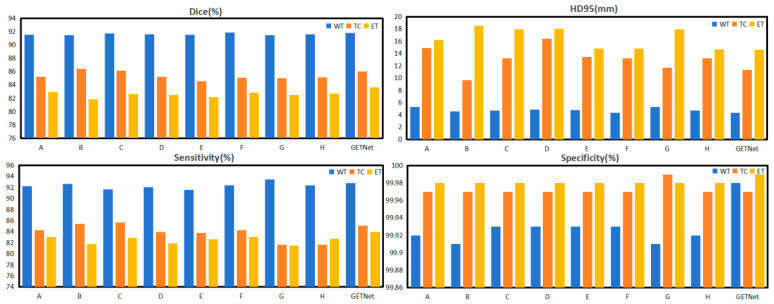
The results of the ablation study of the convex combination in the ECSA module when λ < 1 and λ ≠ 1 − λ.

Table 8 and Figure 12 compare the coefficients of the convex combination in the GNS module; 1 − λ and λ represent the proportion of information processed from cross-attention and self-attention in the VT Decoder Block, respectively. The different proportions of 1 − λ and λ determine which part plays a decisive role, and this has a certain impact on the processing of the later layer. In order to find the optimal combination of λ values in convex combinations, values of λ from 0.1 to 0.9 were tested. The results show that the results for λ = 0.5 and 1 − λ = 0.5 were the best. The results are 91.77, 86.03, 83.64, and 87.15 for WT, TC, ET, and the average Dice coefficient, respectively. It can be seen from the average Dice coefficient that there is indeed a certain improvement when the proportion of cross-attention increases, but this also has little impact on the results. The best effect is achieved when cross-attention and self-attention reach a balance.

**Figure 13:**
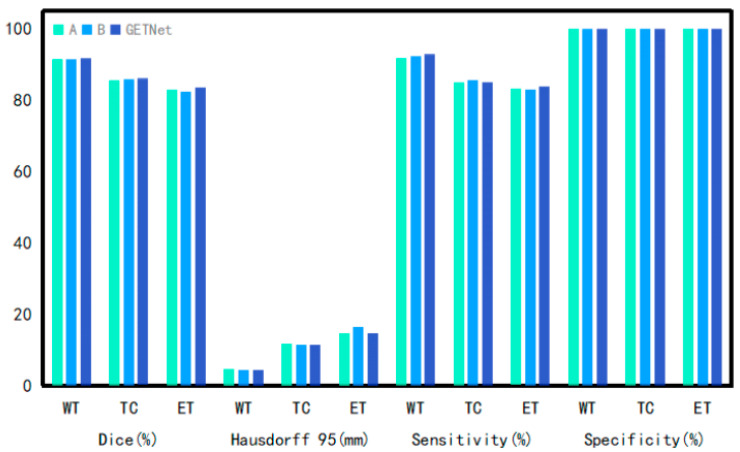
The results of the ablation study of the convex combination in the ECSA module when λ = 1 or λ = 1 − λ.

We also considered the case where convex combinations are not used, meaning the adaptive learning parameters of **ω;** η = 1 and θ = 1 did not exceed the results of λ = 0.5 and 1 − λ = 0.5, as illustrated in Table 9 and Figure 13.

The results are 91.56, 85.54, 82.83, and 86.57 for WT, TC, ET, and the average Dice coefficient, respectively, when η and θ were adaptive learning parameters **ω.** The results were 91.50, 85.76, 82.47, and 86.58 for WT, TC, ET, and the average Dice coefficient, respectively, when η was 1 and θ was 1. The results were 91.77, 86.03, 83.64, and 87.15 for WT, TC, ET, and the average Dice coefficient, respectively, when η was 0.5 and θ was 0.5. The results indicate that further feature processing and extraction are needed to achieve better results when both cross-attention and self-attention contain more information. From the comparison of the results of Table 8 and Table 9, it can be seen that the adaptive learning parameters played a certain role, but the results also need further processing.

#### 4.2.4. Ablation Study of the Frequency Coefficient of FEP in the ECSA Module

**Figure 14:**
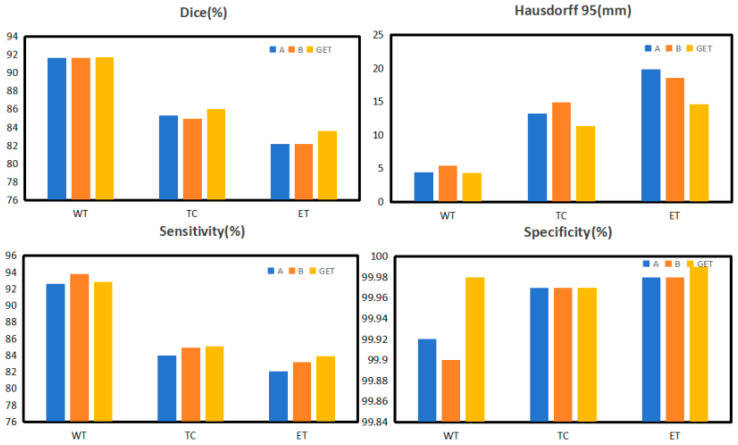
The results of the FEP frequency coefficient in the ECSA module.

Table 10 and Figure 14 show a comparison of the frequency coefficient of FEP in the GNS module. The frequency coefficient of FEP is 10,000 in the case of VT-UNet. In Table 10, Test A represents a frequency coefficient of 5000 in FEP, and Test B represents a frequency coefficient of 20,000. That is, the wavelengths form a geometric progression from 2π to 10,000·λπ. We made an effort to change this coefficient to achieve better results. We conducted experiments to modify the default coefficient by half and to double it. The results indicate that 10,000 is still optimal.

In this section of the experiment, we only performed simple scaling by half or double, and a large number of parameters remained untested. However, from the results in Table 10, it can be seen that the frequency coefficient does not have a particularly significant impact on the final results. Perhaps there will be better results in later testing, but this requires much experimentation.

#### 4.2.5. Comparative Experiment on the Depth-Wise Size of the 3D Patch-Merging Operation

In the original VT-UNet, the depth-wise size does not change as the network deepens, but in the GETNet that we proposed, it does change with depth. Table 11 shows that when the depth-wise size changes with the layers, its performance is not affected, but the floating-point operations per second (FLOPs) are reduced by 48.99G. This indicates that changes in the depth-wise size with the layers can reduce the FLOPs and improve the segmentation efficiency.

## 5. Conclusions

In this paper, we propose GETNet based on VT-UNet, which integrates a GNS block and an ECSA block. It enhances the performance of brain tumor segmentation by effectively fusing local features with long-distance relationships. The GNS module is used between the VT Encoder Block and 3D Patch-Merging Block, improving the shuffle block in ShuffleNetV2, enabling communication of information between different groups of channels, and improving accuracy. We propose the ECSA Block, which works in a bottleneck and can combine the advantages of global and local features, which is beneficial for extracting image feature details. In addition to comparing our results with those of the classic VT-UNet, we compared our results with those of networks based on UNet or Transformer. Our results yield Dice coefficients of 91.77, 86.03, and 83.64, respectively, for three tumor subregions (WT, TC, and ET). Our advantage over architectures based on UNet and Transformer lies in the more effective fusion of local and global features using GNS and ECSA modules. We also conducted ablation experiments on the GNS module, ECSA module, convex combination, and FPE, which proved the effectiveness of our modules. Table 8 shows that for the average Dice coefficient, there is a certain improvement when the proportion of cross-attention increases, but it also has little impact on the results. The best effect is achieved when cross-attention and self-attention reach a balance. From the results in Table 10, it can be seen that the frequency coefficient does not have a particularly significant impact on the final results. Furthermore, quantitative and qualitative experiments demonstrated the accuracy of GETNet. Our architecture and the proposed modules can provide effective ideas for subsequent research.

## Figures and Tables

**Table 1:** Model parameter configuration.

Basic Configuration	Value
PyTorch Version	1.11.0
Python	3.8.10
GPU	NVIDIA RTX A5000 (24 G)
Cuda	cu113
Learning Rate	1.00 × 10^−4^
Optimizer	Adam
Epoch	350
Batch Size	1
Input Size	128 × 128 × 128
Output Size	128 × 128 × 128

**Table 2:** The offline validation results for the comparison of different methods in relation to BraTS2021, with the best performance highlighted in bold.

Methods	Dice (%)			SD			Recall (%)			F1-Score (%)		
	WT	TC	ET	WT	TC	ET	WT	TC	ET	WT	TC	ET
3D U-Net [12]	91.29	89.13	85.78	7.14	15.49	15.93	91.38	88.60	87.31	95.75	95.87	93.16
Att-Unet [60]	91.43	89.51	85.71	9.23	15.15	17.11	90.73	88.68	86.35	96.32	96.43	94.13
UNETR [38]	91.53	88.57	85.27	8.92	15.94	18.48	92.54	88.71	85.92	95.69	95.44	94.15
TransBTS [40]	90.61	88.78	84.29	10.73	16.48	19.30	91.20	87.74	85.73	95.69	96.38	93.35
VT-UNet [37]	92.39	90.12	86.07	8.60	14.48	16.37	92.76	90.61	87.85	96.43	96.06	93.63
Swin Unet3D (2023) [41]	92.85	90.69	86.26	5.67	14.30	17.15	92.18	90.81	87.85	**96.94**	96.42	93.63
GETNet (ours)	**93.04**	**91.70**	**87.41**	**5.53**	**11.60**	**14.01**	**92.87**	**91.36**	**88.22**	96.78	**96.75**	**94.32**

**Table 3:** Ratio (in %) of the improvement in the performance of GETNet compared to different methods. Bold numbers indicate statistical significance (*p* < 0.05).

Methods	WT		TC		ET	
	%Subjects	*p*	%Subjects	*p*	%Subjects	*p*
GETNet (ours) vs. 3D U-Net	**76.4**	**2.987 × 10^−24^**	**82.8**	**6.849 × 10^−18^**	**73.7**	**7.590 × 10^−7^**
GETNet (ours) vs. Att-Unet	**76.1**	**9.093 × 10^−16^**	**81.2**	**4.548 × 10^−14^**	**73.7**	**0.008**
GETNet (ours) vs. UNETR	**76.1**	**1.286 × 10^−6^**	**83.6**	**1.612 × 10^−15^**	**77.2**	**0.092**
GETNet (ours) vs. TransBTS	**78.8**	**4.564 × 10^−24^**	**83.6**	**3.762 × 10^−17^**	**79.6**	**2.579 × 10^−12^**
GETNet (ours) vs. VT-UNet	**73.3**	**2.724 × 10^−6^**	**78.8**	**1.421 × 10^−10^**	**73.7**	**1.576 × 10^−9^**
GETNet (ours) vs. Swin Unet3D	**70.9**	**0.0006**	**76.4**	**0.007**	72.9	0.01

**Table 4:** The results of GETNet when using the LGG/HGG dataset of BraTS2020, with the best performance highlighted in bold.

Methods	Dice (%)			SD			Recall (%)			F1-Score (%)		
	WT	TC	ET	WT	TC	ET	WT	TC	ET	WT	TC	ET
GETNet in HGG cases	**92.74**	**92.53**	**87.24**	4.29	**6.81**	**8.62**	**92.73**	**91.66**	**89.65**	96.41	**96.87**	**92.55**
GETNet in LGG cases	92.63	82.10	77.64	**3.89**	15.86	29.32	91.77	81.87	81.33	**96.82**	92.97	92.03
GETNet in all cases	92.72	90.38	85.26	4.21	10.31	15.84	92.53	89.65	87.94	96.49	96.09	92.44

**Table 5:** The online validation results for the comparison of different methods in relation to BraTS2021, with the best performance highlighted in bold.

Methods	Dice (%)				HD95 (mm)			
	WT	TC	ET	AVG	WT	TC	ET	AVG
3D U-Net [12]	88.02	76.17	76.20	80.13	9.97	21.57	25.48	19.00
Att-Unet [60]	89.74	81.59	79.60	83.64	8.09	14.68	19.37	14.05
UNETR [38]	90.89	83.73	80.93	85.18	4.71	13.38	21.39	13.16
TransBTS [40]	90.45	83.49	81.17	85.03	6.77	10.14	18.94	11.95
VT-UNet [37]	91.66	84.41	80.75	85.60	4.11	13.20	15.08	10.80
E1d3-UNet (2022) [22]	**92.30**	86.30	81.80	86.80	4.34	9.62	18.24	10.73
Orthogonal-Net (2022) [63]	91.40	85.00	83.20	86.53	5.43	9.81	20.97	12.07
SDS-Net (2023) [24]	91.80	**86.80**	82.50	87.00	21.07	11.99	**13.13**	15.40
Swin Unet3D (2023) [41]	90.50	86.60	83.40	86.83	-	-	-	-
QT-UNet-B (2024) [64]	91.24	83.20	79.99	84.81	4.44	12.95	17.19	11.53
Yaru3DFPN (2024) [65]	92.02	86.27	80.90	86.40	**4.09**	**8.43**	21.91	11.48
GETNet (ours)	91.77	86.03	**83.64**	**87.15**	4.36	11.35	14.58	**10.10**

**Table 6:** The results of the ablation study of each module in GETNet, with the best performance highlighted in bold.

Expt	Dice (%)			HD95 (mm)			Sensitivity (%)			Specificity (%)		
	WT	TC	ET	WT	TC	ET	WT	TC	ET	WT	TC	ET
Base	91.66	84.41	80.75	**4.11**	13.20	15.08	-	-	-	-	-	-
Base + GNS	91.50	85.30	81.92	5.36	13.68	20.24	91.67	84.08	82.51	99.93	99.96	99.98
Base + ECSA	91.62	85.82	82.11	4.79	11.44	18.04	91.73	84.99	81.81	99.92	99.97	99.98
Base + GNS + ECSA (GETNet)	**91.77**	**86.03**	**83.64**	4.36	**11.35**	**14.58**	**92.83**	**85.12**	**83.91**	**99.98**	**99.97**	**99.99**

**Table 7:** The results of the ablation study of GN and GeLU in the GNS module, with the best performance highlighted in bold.

Expt	Position	GN	BN	GN + GeLU	BN + ReLU		Dice	HD95	Sen	Spe
A	Unit 1				**√**	WT	91.32	5.72	91.64	99.98
	Unit 2		**√**			TC	84.26	17.18	83.27	99.97
	Unit 3				**√**	ET	83.01	15.02	83.56	99.93
B	Unit 1			**√**		WT	91.36	4.87	92.32	99.92
	Unit 2			**√**		TC	85.89	**10.07**	84.80	**99.97**
	Unit 3			**√**		ET	82.19	19.91	81.90	99.98
C	Unit 1	**√**				WT	91.65	4.67	92.30	99.92
	Unit 2	**√**				TC	85.67	13.69	84.51	99.98
	Unit 3	**√**				ET	82.88	16.71	83.01	99.98
D	Unit 1	**√**				WT	91.42	4.56	**92.87**	99.91
	Unit 2			**√**		TC	85.54	13.10	84.03	99.98
	Unit 3	**√**				ET	82.90	17.90	82.42	99.98
E (GETNet)	Unit 1			**√**		WT	**91.77**	**4.36**	92.83	**99.98**
	Unit 2	**√**				TC	**86.03**	11.35	**85.12**	99.97
	Unit 3			**√**		ET	**83.64**	**14.58**	**83.91**	**99.99**

**Table 8:** The results of the ablation study of the convex combination in the ECSA module when λ < 1 and λ ≠ 1 − λ, with the best performance highlighted in bold.

Expt	λ	1 − λ	Dice (%)			HD95 (mm)			Sensitivity (%)			Specificity (%)		
			WT	TC	ET	WT	TC	ET	WT	TC	ET	WT	TC	ET
A	0.1	0.9	91.56	85.22	82.94	5.28	14.91	16.22	92.22	84.26	83.08	99.92	99.97	99.98
B	0.2	0.8	91.46	**86.41**	81.86	4.56	**9.69**	18.52	92.65	85.39	81.71	99.91	99.97	99.98
C	0.3	0.7	91.73	86.18	82.61	4.68	13.2	17.96	91.65	**85.65**	82.85	99.93	99.97	99.98
D	0.4	0.6	91.63	85.23	82.47	4.86	16.44	18.01	92.07	83.97	81.91	99.93	99.97	99.98
E	0.6	0.4	91.50	84.57	82.16	4.75	13.46	14.81	91.56	83.82	82.66	99.93	99.97	99.98
F	0.7	0.3	**91.89**	85.08	82.84	**4.33**	13.26	14.83	92.4	84.3	83.05	99.93	99.97	99.98
G	0.8	0.2	91.48	85.02	82.51	5.28	11.71	17.97	93.43	81.65	81.48	99.91	99.99	99.98
H	0.9	0.1	91.58	85.13	82.72	4.69	13.21	14.7	92.39	81.64	82.71	99.92	99.97	99.98
GETNet	**0.5**	**0.5**	91.77	86.03	**83.64**	4.36	11.35	**14.58**	**92.83**	85.12	**83.91**	**99.98**	**99.97**	**99.99**

**Table 9:** The results of the ablation study of the convex combination in the ECSA module when λ = 1 or λ = 1 − λ, with the best performance highlighted in bold.

Expt	η	θ	Dice (%)			HD95 (mm)			Sensitivity (%)			Specificity (%)		
			WT	TC	ET	WT	TC	ET	WT	TC	ET	WT	TC	ET
A	**ω**	**ω**	91.56	85.54	82.83	4.67	11.70	14.78	91.82	84.91	83.10	99.93	99.97	99.98
B	1	1	91.50	85.76	82.47	4.49	11.39	16.44	92.28	**85.72**	83.08	99.92	99.97	99.98
GETNet	0.5	0.5	**91.77**	**86.03**	**83.64**	**4.36**	**11.35**	**14.58**	**92.83**	85.12	**83.91**	**99.98**	**99.97**	**99.99**

**Table 10:** The results of the FEP frequency coefficient in the ECSA module, with the best performance highlighted in bold.

Expt	λ	Dice (%)			HD95 (mm)			Sensitivity (%)			Specificity (%)		
		WT	TC	ET	WT	TC	ET	WT	TC	ET	WT	TC	ET
A	5000	91.70	85.33	82.17	4.47	13.23	19.87	92.63	83.96	82.07	99.92	99.97	99.98
B	20,000	91.64	84.96	82.21	5.38	14.97	18.57	**93.79**	84.95	83.18	99.90	99.97	99.98
GETNet	10,000	**91.77**	**86.03**	**83.64**	**4.36**	**11.35**	**14.58**	92.83	**85.12**	**83.91**	**99.98**	**99.97**	**99.99**

**Table 11:** The results of a comparative experiment on the depth-wise size of the 3D Patch-Merging operation, with the best performance highlighted in bold.

Expt	Dice (%)			HD95 (mm)			Sensitivity (%)			Specificity (%)			FLOPs
	WT	TC	ET	WT	TC	ET	WT	TC	ET	WT	TC	ET	
A	91.50	**86.83**	82.88	4.45	**9.65**	17.88	92.09	**86.58**	82.54	99.92	99.96	99.97	130.94G
GETNet	**91.77**	86.03	**83.64**	**4.36**	11.35	**14.58**	**92.83**	85.12	**83.91**	**99.98**	**99.97**	**99.99**	**81.95G**

## Data Availability

Datasets released to the public were analyzed in this study. The BraTS2021 dataset can be found through the following link: https://www.med.upenn.edu/cbica/brats2021/#Data2 (accessed on 12 June 2024).
